# Peri-Elastodynamic Simulations of Guided Ultrasonic Waves in Plate-Like Structure with Surface Mounted PZT

**DOI:** 10.3390/s18010274

**Published:** 2018-01-18

**Authors:** Subir Patra, Hossain Ahmed, Sourav Banerjee

**Affiliations:** Integrated Material Assessment and Predictive Simulation Laboratory, Department of Mechanical Engineering, University of South Carolina, Columbia, SC 29208, USA; spatra@email.sc.edu (S.P.); hahmed@email.sc.edu (H.A.)

**Keywords:** lamb wave, peridynamic, peri-elastodynamics, frequency-wavenumber, wave propagation, nondestructive evaluation (NDE), ultrasonics, computational NDE, structural health monitoring (SHM)

## Abstract

Peridynamic based elastodynamic computation tool named Peri-elastodynamics is proposed herein to simulate the three-dimensional (3D) Lamb wave modes in materials for the first time. Peri-elastodynamics is a nonlocal meshless approach which is a scale-independent generalized technique to visualize the acoustic and ultrasonic waves in plate-like structure, micro-electro-mechanical systems (MEMS) and nanodevices for their respective characterization. In this article, the characteristics of the fundamental Lamb wave modes are simulated in a sample plate-like structure. Lamb wave modes are generated using a surface mounted piezoelectric (PZT) transducer which is actuated from the top surface. The proposed generalized Peri-elastodynamics method is not only capable of simulating two dimensional (2D) in plane wave under plane strain condition formulated previously but also capable of accurately simulating the out of plane Symmetric and Antisymmetric Lamb wave modes in plate like structures in 3D. For structural health monitoring (SHM) of plate-like structures and nondestructive evaluation (NDE) of MEMS devices, it is necessary to simulate the 3D wave-damage interaction scenarios and visualize the different wave features due to damages. Hence, in addition, to simulating the guided ultrasonic wave modes in pristine material, Lamb waves were also simulated in a damaged plate. The accuracy of the proposed technique is verified by comparing the modes generated in the plate and the mode shapes across the thickness of the plate with theoretical wave analysis.

## 1. Introduction

In Structural Health Monitoring (SHM) research, Lamb waves are widely used for damage detection in the metallic plate-like structures [1,2]. High-frequency ultrasonic actuators and sensors are strategically mounted on the plate-like structure to detect, localize and characterize the damages [3]. Symmetric (S_0_) and Antisymmetric (A_0_) Rayleigh-Lamb wave modes while travel through the plate, interacts with the boundaries and the discontinuities [4] and are subjected to mode conversion. Efficient diagnostic and prognostic algorithms are then employed to estimate the severity of the damage and the damage growth.

Sensor signals play a critical role in quantifying the extent of damage within the structure. In most practical cases with SHM, the damage state of the material is unknown and the sensor signals are the observables. There could be infinite possibilities of damage states in the material and it is impossible to experimentally obtain the understanding of the sensor signals due to the varying damage states. Hence, for SHM, an offline simulation tool will add tremendous value [5] to the understanding of the physics of the wave propagation and its interaction with the damages. Unlike experiments, in simulations, various host structure geometries and different damage scenarios could be analyzed more inexpensively. Thus recently, Computational NDE and SHM [6,7,8] have gained enormous popularity. Existing analytical approaches are insufficient to simulate the wave propagation in three-dimensional structures with complex geometries and boundary conditions. Thus, a number of numerical techniques such as spectral finite element method (SEM) [9,10,11], finite element method (FEM) [12], boundary element method (BEM) [13], mass-spring lattice model (MSLM) [14], finite difference method (FDM) [15], finite strip method [16,17], cellular automata [18,19], elastodynamic finite integration technique (EFIT) [20] were developed. While these techniques can predict the sensor signals with a considerable accuracy, fine discretization in spatial and time domains makes them computationally expensive. To overcome this issue, a few semi-analytical techniques, such as distributed point source method (DPSM) [21], local interaction simulation approach (LISA) [7,22,23] and semi-analytical finite element (SAFE) [24] methods were developed to reduce computational burden. DPSM is a meshless semi-analytical method which requires displacement and stress Green’s functions in the problem formulation. It was found that the frequency domain DPSM is a much faster method than the FEM, BEM, SEM, etc. Moreover, DPSM is more accurate than the FEM, while it avoids the inherent issue with the spurious reflection [21]. Among the time domain approaches, LISA is similar to the EFIT method, requires additional local interaction of material points in time and space domain. This makes them computationally expensive. A parallel computing facility would be necessary for a problem similar to the one presented in this article. Additional advantages and disadvantages of these techniques can be found in Ref. [25]. In this article, a newly formulated technique, Peri-elastodynamics is proposed as an alternative approach to simulate the wave propagation in three dimension (3D). The objective of this article is to present the Peri-elastodynamic formulation for the 3D ultrasonic wave simulation and demonstrate that the guided ultrasonic waves can be accurately simulated using peridynamic theory.

The reason for proposing a new method herein, is that by changing the boundary conditions virtual wave propagation can be studied while the material is still under operation or loading. Complementary to the existing methods like DPSM, EFIT, LISA, SEM, Peri-elastodynamics can be used to predict both the damage growth as well as the wave propagation signals, simultaneously. In Peri-elastodynamic, damage detection and characterization can be performed while the damage is still growing, without altering any meshing or discretization keeping the same parent model. This would not only be impossible using Finite Element Method (FEM) but would be equally impossible by the newer models like DPSM, EFIT, LISA, SEM. Hence, Peri-elastodynamic would be advantageous over the existing wave simulation tools. Using the proposed Peri-elastodynamic simulation, when the damage grows under operation, only the damage matrix can be modified. Damage propagation in metallic and composite structures [26,27] were successfully presented by the earlier researchers using peridynamics. Similarly, two dimensional (2D) in plane wave propagation were also simulated [17,18,28,29] using peridynamic theory. However, no work has been reported so far to simulate the 3D Lamb wave modes in a plate-like structure that could be used for simulating virtual NDE and SHM experiments.

Peridynamic theory (PD) was developed by Silling et al. [26]. In the fundamental equation of motion of a body, the peridynamic formulation employs the integral of the force density instead of the divergence of the stress tensor. The integral approach makes it suitable to simulate the damage propagation problem [30] without altering the mesh. Ha and Bobaru [31] studied dynamic crack propagation and crack branching in glass under dynamic loading. Madenci and Oterkus [32] employed PD theory to predict the crack propagation in a composite and metallic plate. However, only a few articles can be found on the application of PD to solve the wave propagation problems. Nishawala et al. [18] recently used bond-based peridynamic theory to simulate Rayleigh wave propagation in a 2D isotropic (CR-39) plate. In this work, a ramp loading with a short time pulse was used to displace the material points located at one end of the plate to generate the surface skimming Rayleigh wave. Only recently, Hafezi et al. [28,33,34] employed PD theory to model the in plane longitudinal ultrasonic wave in an aluminum plate [17,29]. In these works, the elastic wave propagation was simulated by considering only one layer of the material points without out of plane deformation. Hence, the method is inefficient and insufficient to simulate the Lamb wave modes. Additionally, comparing the peridynamic and continuum formulation of strain energy density, the mathematical equation for the bond constant used in these articles [28,33,34] is incorrect, which is correctly presented in this article. Studies reported were neither validated with any analytical solution nor compared with any experimental results. In this article, it is argued with corrected bond constant that one layer of the material point is not only insufficient but also inaccurate to simulate the Lamb wave modes. Monolayer simulation cannot accommodate the out of plane deformation. Therefore, in this article, it is shown that with specific discretization schemes, at least three layers of spatial material points are required to correctly capture the fundamental Lamb wave modes (S_0_ and A_0_) in a plate.

In addition to the SHM of plate structure, NDE is a crucial step for the ensured functionality and fail-safe design of specific plate-like MEMS devices. Such virtual NDE experimental scenarios for MEMS devices could be simulated using Peri-elastodynamic as proposed in this article. From here onwards in this article, the plate-like structure for SHM and the plate-like structure in MEMS devices for their characterization are commonly termed as plate-like structure or material.

## 2. Peri-Elastodynamic Formulation

### 2.1. Basics of Bond-Based Peridynamic Formulation

**Figure 1:**
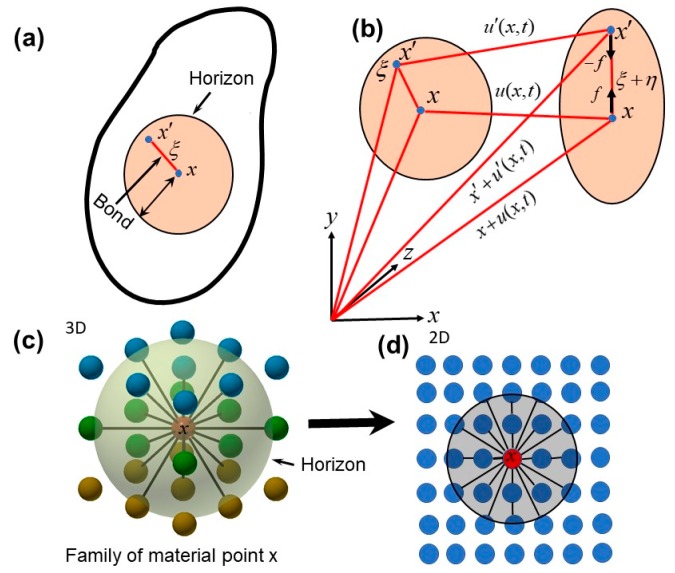
Kinetics of peridynamics deformation [35,36]: (**a**) Horizon, bond and family of a material point x in the reference configuration, (**b**) Deformed configuration, (**c**) Illustration of interactions of material points within a family in three-dimension, (**d**) Interactions of material points in two-dimension.

Peridynamic is a meshless simulation method where a material body is discretized into a series of material points. To illustrate the kinetics, an undeformed and a deformed state of two particles are shown in Figure 1a,b, respectively. The deformation between two material points produces a pairwise interaction along the bond (Figure 1b). The equation of equilibrium at the material point x at time *t* can be written as follows [26],

(1) $$\rho \ddot{u}(x,t)=\int_{H}f(u(x^{\prime },t)-u(x,t),x^{\prime }-x)dV^{\prime }+b(x,t),$$

where, u, ρ, b, f and $V^{\prime }$ are the displacement, density, body force per unit volume at the material point, the pairwise force acting along the bond between x and $x^{\prime }$ and the volume of the material point at $x^{\prime }$, respectively. The integral of the forces acting at the parent material point x is performed over a finite region H, called Horizon in the peridynamic theory. The material points inside a Horizon are called the family members of the parent material point. The parent material point interacts with the other material points within its family while the interactions with the points outside the Horizon are negligible. Figure 1c,d represent a family of a material point in three-dimension and in two-dimension, respectively. 

Relative distance and the displacement between the two material points in the reference configuration (Figure 1a) can be viz.,

(2) $$\xi =x^{\prime }-x$$

(3) $$\eta =u(x^{\prime },t)-u(x,t)$$

Relative displacement between the two material points in the deformed configuration can be expressed as (Figure 1),

(4) $$\left(\xi +\eta )=(u(x^{\prime },t)+x^{\prime })-(u(x,t)+x\right)$$

Constitutive law in the peridynamic approach is expressed as follows,

(5) $$f(\eta ,\xi )=c(\xi )s\frac{\xi +\eta }{\left\|\xi +\eta \right\|}$$

where, c and s are the bond constant and the stretch of the bond, respectively. The bond constant for a two-dimensional material body is calculated by balancing the strain energy density from the continuum mechanics and peridynamic formulation viz. [32],

(6) $$c\left(\xi \right)=9E/\left(2{\pi h\delta }^{3}\right)$$

where E, h and δ are the Young’s Modulus, the half thickness of the plate and the radius of the Horizon H, respectively. In peridynamics, the stretch is the ratio of the change in the length of the peridynamic bond due to the deformation of the initial bond length, expressed as follows,

(7) $$s=\frac{\left|\xi +\eta \right|-\left|\xi \right|}{\left|\xi \right|}$$

### 2.2. Actuator Modelling with in Plane Excitation

**Figure 2:**
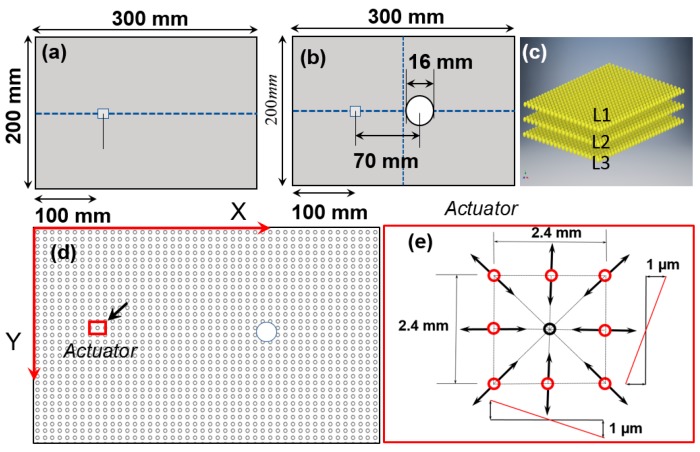
The schematics showing the geometry of an Aluminum 6061-T6 plate used in the simulation: (**a**) Pristine plate with PZT mounted on the top surface, (**b**) Plate with a through-thickness hole and a PZT patch, (**c**) Discretization of the plate and material layers (top, middle and bottom surfaces, L1, L2 and L3, respectively), (**d**) Schematics of Peri-elastodynamics discretization of the plate showing PZT and the hole (**e**) Boundary condition: Particle displacement due to the PZT excitation.

**Figure 3:**
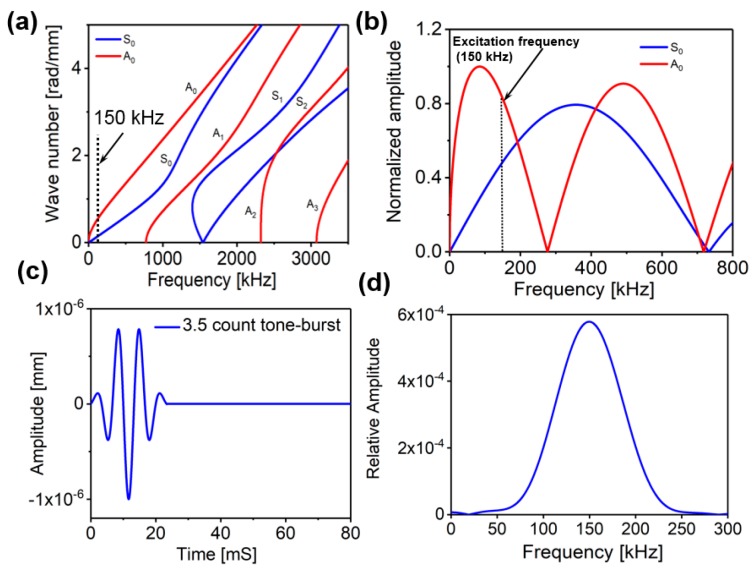
(**a**) Dispersion curves for 2 mm thick Aluminum 6061-T6 plate, (**b**) Tuning curve of an Aluminum 6061-T6 plate (2 mm thickness) with a standard 7 mm PZT, (**c**) 3.5 count tone burst signal (displacement input signal) with 150 kHz central frequency shown in time domain, (**d**) Frequency domain representation of the excitation signal.

In SHM, PZT actuator is attached to the host structure as shown in Figure 2a. In this article, a 300 mm × 200 mm plate is used for the wave propagation simulation (Figure 2a). A plate with a hole is also considered to study the wave damage interactions (Figure 2b). Lamb waves are generated by applying a standard tone-burst voltage signal to the PZT actuator [3]. Except the interface between the PZT actuator and the plate, all other boundaries of the plate are considered stress-free in the simulation. The voltage signal actuates the PZT and transformed the energy into in plane mechanical strain. The in-plane strain causes the rapid localized displacement in the host structure, which results in the Lamb wave propagation in the plate. The Lamb wave modes create out of plane displacements and hence, to accommodate such deformation three layers of material points are used in the modeling (Figure 2c). Figure 2d shows the discretization used in this study which is further described in Section 2.4. Displacement in the structure varies linearly along the length of the PZT and attains a maximum value at the boundaries [37] as shown in Figure 2e. In this study, a square PZT with a dimension of 2.4 × 2.4 mm is modeled by applying a maximum of 1 μm in plane radial displacement to the circumferential material points, shown in the Figure 2e. The displacements of the material points at the center of the PZT were enforced to zero. The equation for variation of displacement due to the application of a tone burst signal is expressed as.

(8) $$u\left(x,t\right)=U\left(x\right)e^{-{\mathrm{pt}}^{2}/2}\sin\left(\omega_{c}t\right)$$

where, $\omega_{c}$ and U(x) are the central frequency and the maximum displacement amplitude of the excitation signal given to different material points, respectively. The parameter p in the Equation (8) is expressed as

(9) $$p={\left(2{\mathrm{kh}\omega }_{c}/N_{c}\right)}^{2}$$

where, k, h and $N_{c}$ are the signal shape factor, the half thickness of the specimen and the number of cycles of the actuation signal, respectively. To select the desired excitation frequency, the tuning curves for S_0_ and A_0_ modes in the Aluminum 6061-T6 were obtained from an open source software “Waveform Revealer” [38], developed by the LAMSS laboratory at the University of South Carolina (USC). As shown in Figure 3a,b, the central frequency of 150 kHz is chosen to make sure that only the S_0_ and A_0_ modes are excited when the modal amplitudes are comparable but nonequal. In the present study, a 3.5 count tone-burst signal with the central frequency ($\omega_{c}$) of 150 kHz is used. Figure 3c,d show the time domain signal and its frequency content, respectively.

### 2.3. Numerical Time Integration

In Peri-elastodynamics approach, the plate is spatially discretized into a finite number of material points. Each material point has finite volume in the reference configuration. Material volume for 3D uniform discretization grid is calculated as $dl^{3}$, where dl is the element length [32,39]. By replacing the integration with a finite summation over all the material points inside the Horizon, the equation of motion at material point *i* after the time step *n* can be expressed as,

(10) $$\rho {\ddot{u}}_{i}^{n}=\sum_{N_{f}}f(u_{f}^{n}-u_{i}^{n},x_{f}-x_{i})V_{f}+b_{i}^{n}$$

The net force (f) acting on a material point is calculated by summing the peridynamic forces on the parent material point due to all the neighboring points inside its Horizon. $N_{f}$ represents the number of material points within the Horizon enclosing the parent material point *i*. The Velocity-Verlet integration [31] scheme is employed in this study to calculate the displacements in the time domain for given boundary and initial conditions as follows,

(11) $$v_{i}^{n+1/2}=v_{i}^{n}+\frac{\Delta t}{2\rho_{i}}f_{i}^{n}\;u_{i}^{n+1/2}=u_{i}^{n}+\frac{\Delta t}{2\rho_{i}}v_{i}^{n+1/2}\;v_{i}^{n+1}=v_{i}^{n+1/2}+\frac{\Delta t}{2\rho_{i}}f_{i}^{n+1}$$

Stability of the numerical solution can be obtained for a small-time step Δt and a spatial discretization step Δs. To have a convergence of the displacements, the detailed procedure to select the time step (Δt) and spatial discretization (Δs) is discussed in Section 2.4.

### 2.4. Peri-Elastodynamic Spatial and Temporal Discretization

Proper spatial and temporal discretization are the critical parameters for the convergence of the solution in wave propagation simulation. Maximum spatial discretization (Δs) must meet the criterion below [40],

(12) $$\lambda_{min}=\frac{c_{min}}{f};\;\Delta s=\lambda_{min}/10$$

where, $\lambda_{\min}$ is the minimum wavelength of the Lamb wave modes and $c_{\min}$ is the minimum phase velocity of the simulated modes at the excitation frequency f. Phase velocity at the excitation frequency can be easily obtained from the theoretical dispersion curves. In this work, the phase velocity of the $A_{0}$ mode at 150 kHz is used as $c_{\min}$ in Equation (12). The Courant–Friedrichs–Levy condition is used to obtain a numerically stable time step (Δt) [40].

(13) $$\Delta t=\frac{\Delta s}{c_{max}\sqrt{3}}$$

where, $c_{\max}$ is the maximum phase wave velocity of the propagating modes. In this work, the phase velocity of the S_0_ mode at 150 kHz is the $c_{\max}$ in Equation (13). Thus, to obtain a converging solution, the spatial and the temporal step sizes are chosen to be 1.2 mm and 0.01 μS, respectively, satisfying the Equations (12) and (13).

Material points are chosen in a grid fashion with a spacing of Δs to model the plate with a layer spacing of 1 mm between each layer L1, L2 and L3. 41,750 material points were used in each layer in the pristine plate. A total of 125,250 material points was used in the simulation including all the three layers L1, L2 and L3. In case of the damaged plate (with hole), there was 41,610 material points in each layer and a total of 124,830 material points were used for the Peri-elastodynamic simulation. Each parent point is assigned with a family based on its Horizon and bonds were established between each pair of material points within the family. A 3.015ΔS was used as the Horizon size in the simulation. To model a plate with a through-thickness hole, a pristine discretization is performed and then the material points are removed from the geometry to produce the hole.

## 3. Lamb Wave Dispersion Relation

Dispersion curves of various Lamb wave modes are used to predict the existence of various modes at a particular excitation frequency [3]. Two types of Lamb wave modes exist in a plate based on the particle motion, named Symmetric modes (i.e., S_0_, S_1_, S_2_…_)_ and Antisymmetric modes (i.e., A_0_, A_1_, A_2_…). Generation of the Lamb wave modes in a plate depends on the frequency of excitation, the thickness of the plate and the material properties (Density, Young’s modulus or Shear Modulus and Poisson’s ratio) of the material. Dispersion of various Lamb wave modes is obtained by solving Rayleigh-Lamb wave equations. Rayleigh-Lamb wave equation for symmetric Lamb wave modes is expressed by,

(14) $$\frac{tan(qh)}{tan(ph)}=-\frac{4k^{2}qp}{{\left(k^{2}-q^{2}\right)}^{2}}$$

For antisymmetric modes, equation is written as follows,

(15) $$\frac{tan(qh)}{tan(ph)}=-\frac{{\left(k^{2}-q^{2}\right)}^{2}}{4k^{2}qp}$$

Parameters in the above equations are expressed as,

(16) $$p^{2}=\frac{\omega^{2}}{c_{L}^{2}}-k^{2}\;;\;q^{2}=\frac{\omega^{2}}{c_{S}^{2}}-k^{2};\;c_{L}=\sqrt{\frac{2\mu (1-\nu )}{\rho (1-2\nu )}};\;c_{s}=\sqrt{\frac{\mu }{\rho }}$$

where ω, k, $c_{s}$, $c_{L}$, ν, μ, ρ and H are the angular frequency, wavenumber, the shear wave velocity, the longitudinal wave velocity, the poison’s ratio, the shear modulus, the density and the half thickness of the plate, respectively. In this work, the dispersion curves for various Lamb wave modes in an Aluminum 6061-T6 plate were calculated using the commercially available ‘Disperse’ software [41], designed by the Imperial College, London, UK, as shown in Figure 3a. The plate thickness was set to 2 mm and the material properties were set to the values listed in Table 1.

## 4. Numerical Computation and Results

The Peri-elastodynamic simulations were performed on a workstation with two Intel Xeon (R) CPU E5-2650 V3 2.30 GHz processors with total 128 GB RAM, in a single core. One simulation was completed within a reasonable time of ~46.5 h. Note that this problem is highly parallelizable and can be implemented with distributed clusters, GPUs, multiple threads, or a combination of these methods. Preliminary translation of this MATLAB program into multithreaded C++ resulted in a 20 times speedup.

### 4.1. Lamb Wave Propagation in the Pristine Plate

**Figure 4:**
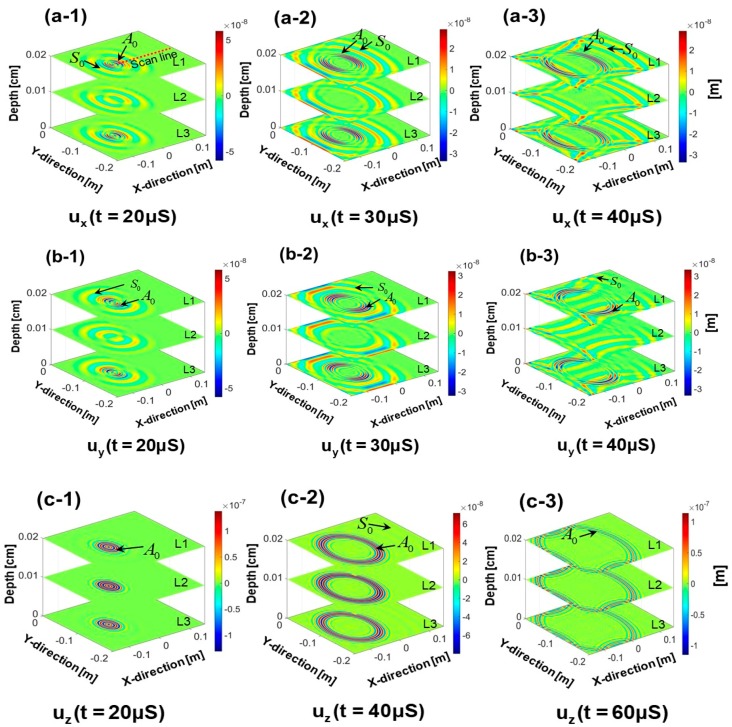
Time domain in plane and out of plane displacement waveform: (**a**) $u_{x}(x,y,t)$ at t = 20, 30 and 40 μs, (**b**) $u_{y}(x,y,t)$ at t = 20, 40 and 60 μs, (**c**) $u_{z}(x,y,t)$ at t = 20, 40 and 60 μs.

In this article, fundamental Lamb wave modes (S_0_ and A_0_) are simulated which are widely used in the damage detection with ultrasonic SHM. Figure 4 shows the out of plane displacements $u_{z}(x,y,t)$ and, in plane displacements $u_{x}(x,y,t)$ and $u_{y}(x,y,t)$ for the Lamb wave propagation plotted at t = 20, 40 and 60 μS, respectively. Each stack of the figure is plotted with the respective displacement pattern in the layers L1, L2 and L3. It can be seen that the S_0_ mode travels faster than the A_0_ mode and confirms the dispersion curves. In wave propagation simulation, most inaccuracy comes from the boundary reflections if the results are not converged. The best approach to judge if the simulation of the wave propagation is converged is to evaluate the boundary reflections. In the present simulation, the reflected modes from the plate boundaries are clearly visible in the figures. Usually, there is a possibility of divergence of the solution at the boundaries due to the nonconvergence of the solution. However, with the specific steps with the Peri-elastodynamic process described in this paper, the results will be converged and bounded boundary reflections will be achieved. 

In the top (L1) and the bottom (L3) layers in Figure 4(a-1–a-3) and Figure 4(b-1–b-3), both Symmetric and Antisymmetric modes (S_0_ and A_0_) are visible in the wave fields composed of in plane displacements. The contribution of the A_0_ mode in the in-plane motion at the middle layer (L2) of the plate is negligible but the out of plane motion is dominant. As shown in Figure 4(c-1–c-3), the out of plane displacement of the A_0_ mode is visible (i.e., contribution from $u_{z}(x,y,t)$) while the S_0_ mode is barely noticeable. This is because the displacements of the in-plane particles of the S_0_ mode dominates over the out of plane motion of the particles. Also, the contribution of the S_0_ mode in the displacement of the middle layer (L2) of the plate is negligible, because, in the S_0_ mode, the middle layer (L2) remains undisturbed. 

**Figure 5:**
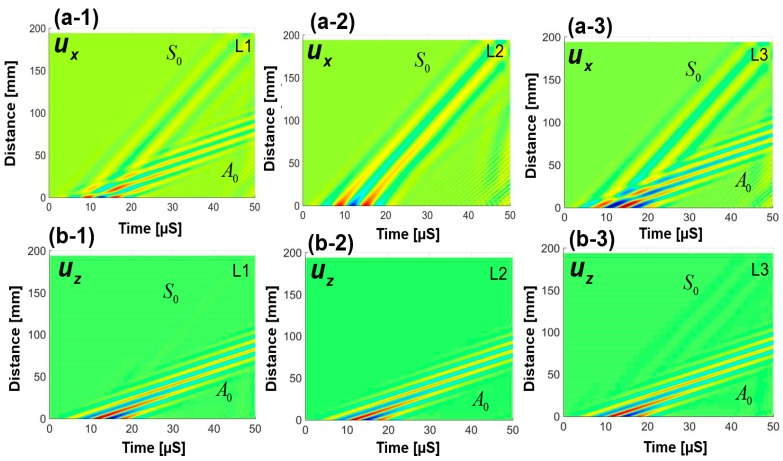
Space-time in plane and out of plane displacement fields: (**a-1**) $u_{x}(x,t)$ at the top (L1), (**a-2**) $u_{x}(x,t)$ at the middle layer (L2), (**a-3**) $u_{x}(x,t)$ at the bottom layer (L3), (**b-1**) $u_{z}(x,t)$ at the top layer (L1), (**b-2**) $u_{z}(x,t)$ at the middle layer (L2), (**b-3**) $u_{z}(x,t)$ at the bottom layer (L3).

Time-space representation of the in plane ($u_{x}(x,t)$) and the out of plane displacement ($u_{z}(x,t)$) are presented in Figure 5. Displacements at the top, middle and the bottom surfaces (L1, L2 and L3), are presented to investigate the existence of the different Lamb wave modes and their contribution to the displacement in each layer. In Figure 5(a-1–a-3), it is observed that the S_0_ mode contributes to the in-plane displacement in all layers and the A_0_ mode contributes only to the top and the bottom layers. Time-space representation of the out of plane displacement is shown in Figure 5(b-1–b-3), which show that the A_0_ mode had a higher amplitude than the S_0_ mode. A_0_ mode contributed to the displacement of all the layers (L1, L2 and L3), whereas, S_0_ mode contributed only to the top (L1) and the bottom (L3) layers. Video S1 in the supplementary material shows the guided wave propagation through the $u_{z}(x,y,t)$ displacement of the layer L1 of the pristine state of the plate used in this article.

### 4.2. Vector Field Representation of the Lamb Wave Modes

**Figure 6:**
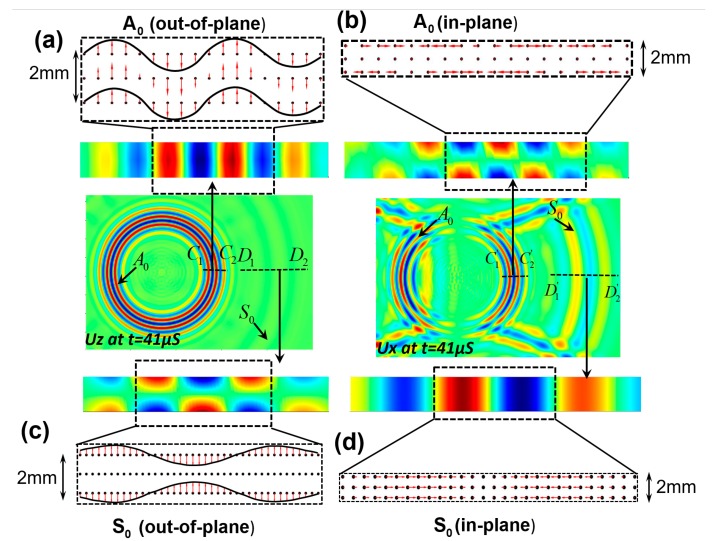
Peri-elastodynamics simulation, vector field and displacement distribution of the S_0_ and A_0_ modes across the thickness of the plate: (**a**) Vector field of the A_0_ mode for out of plane motion, (**b**) Vector field of the A_0_ mode for in plane motion, (**c**) Vector field of the S_0_ mode for out of plane motion, (**d**) Vector field of the S_0_ mode for in plane motion.

To prove the accuracy of the Peri-elastodynamic simulation, the characteristics of the Lamb wave modes (S_0_ and A_0_), in plane and out of plane particle motion across-the-thickness are plotted in Figure 6, after 41 μS. Out of plane ($u_{z}(x,y,t)$) and in plane ($u_{x}(x,y,t)$) displacement distribution of A_0_ mode are extracted along the cross-sections $C_{1}-C_{2}$ and $C_{1}^{\prime }-C_{2}^{\prime }$, of the plate. Similarly, the out of plane and the in-plane displacement distribution of S_0_ mode is plotted along the cross-section lines $D_{1}-D_{2}$ and $D_{1}^{\prime }-D_{2}^{\prime }$, respectively. Vector fields and displacement distributions are shown in Figure 6a–d. It is observed in Figure 6a that all particles moved either upwards (+Z) or downwards (−Z) with variable amplitude (like bending motion) due to the generation of the A_0_ mode. In Figure 6c, the top and the bottom layers are symmetrically displaced with respect to the mid-plane and the displacement of the mid-plane is almost zero due to the generation of the S_0_ mode. In plane particle motion in A_0_ and S_0_ modes are also shown in Figure 6b,d. In Figure 6b, the particles at the top and the bottom layers are moved in the opposite directions along the in-plane direction and the displacement of the mid-plane is zero due to the generation of the A_0_ mode. In Figure 6c, the particle displacements are constant across the thickness due to the S_0_ mode. Vector fields and the mode shapes in Figure 6 indicate that the Peri-elastodynamics simulated the Lamb waves accurately. 

## 5. Analysis of the Sensor Signals

### Frequency-Wavenumber Analysis: Verification of the Simulation Results

Multidimensional Fourier transform is widely used to separate the different Lamb wave modes [42]. Two-dimensional and three-dimensional Fourier transforms (2D or 3D FFT) are performed on space and time domain data. The equation that transforms the time-space wavefield data into the frequency-wavenumber representation of the wave field and can be expressed as follows [42],

(17) $$u_{p}\left(k_{j},\omega \right)=\int_{-\infty }^{\infty }\int_{-\infty }^{\infty }\int_{-\infty }^{\infty }u_{p}\left(x_{j,}t\right)e^{-2\pi i\left(k.x\right)}e^{-i\omega t}dx_{1}dx_{2}dt$$

where, j take the values, 1 and 2, p take values 1, 2 and 3, $u_{p}\left(x_{j,}t\right)$ designates the p-th displacement after time t at the point $x_{j}$ located on the 2D x-y plane. $u_{p}\left(k_{j},\omega \right)$ designates the p-th displacement at frequency ω in reciprocal space of wavenumbers at the point $k_{j}$ located on the 2D reciprocal $k_{x}-k_{y}$ plane. Here index, 1, 2 and 3 stands for the coordinate x, y and z.

In multi-modal wave propagation analysis, distinguishing the different modes from a time domain signal is difficult, especially on a small plate where the wave modes tend to overlap. In this work, frequency-wavenumber plots are presented to visualize the different modes separately. This is also verified by comparing the simulated dispersion results with the theoretical dispersion curves. For this purpose, 2D and 3D Fast Fourier Transforms (FFT) were performed on the simulated displacement wave field to obtain the frequency-wavenumber representations. 

To perform the 2D-FFT, out of plane ($u_{z}(x,y,t)$) and in plane ($u_{x}(x,y,t)$), displacement data are obtained across-the-thickness of the plate along the selected red dotted line shown in Figure 4(a-1). 163 spatial points with a resolution of 1.2 mm along the red line shown in Figure 4(a-1) were used in the analysis. Matrix size used to store the displacements wave field was 163 × 8000. Note that, the 2D FFT was performed on the displacement vectors obtained from all the three material layers (L1, L2 and L3). 

**Figure 7:**
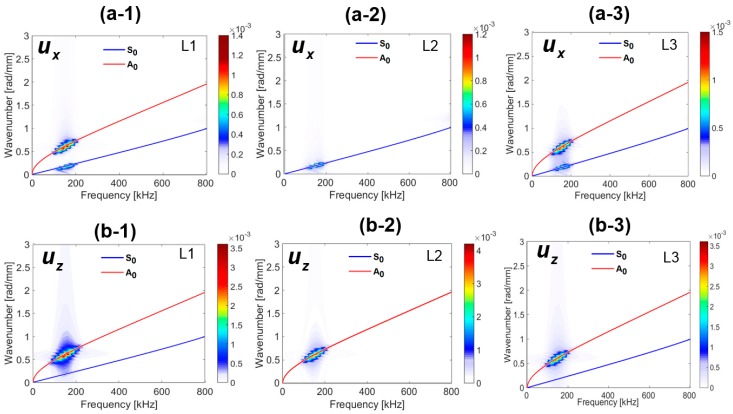
Frequency-wavenumber (FW) representation of the displacement field at the pristine state: (**a-1**) FW of the in plane displacement at the top surface (L1), (**a-2**) FW of the in plane displacement at the mid-surface (L2), (**a-3**) FW of the in plane displacement at the bottom surface (L3), (**b-1**) FW of the out of plane displacement at the top surface (L1), (**b-2**) FW of the out of plane displacement at the mid-surface (L2), (**b-3**) FW of the out of plane displacement at the bottom surface (L3).

Frequency-wavenumber domain representation of the in-plane displacement ($u_{x}(x,y,t)$) is depicted in Figure 7(a-1–a-3), respectively. Both the S_0_ and A_0_ modes are identified at the top and the bottom material layers. This is because they both significantly contributed to the energy of the in-plane wave motion. The amplitudes of the A_0_ mode are slightly greater than that of the S_0_ mode. A similar phenomenon is predicted from the tuning curve of the plate at 150 kHz. The contribution of the in-plane motion of the A_0_ mode to the energy of the middle layer is almost zero. Similarly, Figure 7(b-1–b-3) are obtained from the wavenumber-frequency domain representation of the out of plane ($u_{z}(x,y,t)$) displacements at the top (L1), middle (L2) and the bottom layer (L3), respectively. The energy distribution of the A_0_ mode is higher than the S_0_ mode at all the material layers. The S_0_ mode is visible only at the top and the bottom layers of very low amplitude. This is because the out of plane motion of the particles in S_0_ mode is very low and the displacement at the midplane is almost zero.

**Figure 8:**
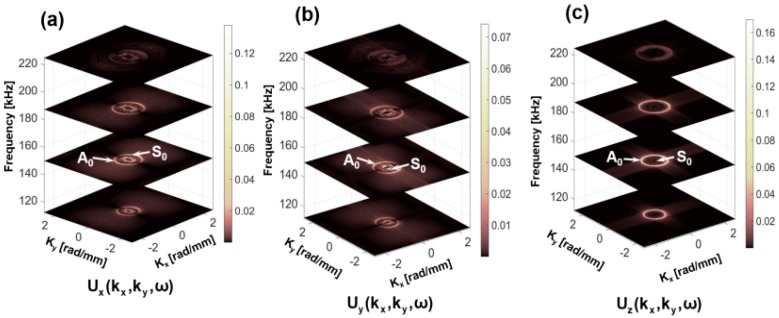
3D Fourier transform of the in plane and the out of plane displacement at the top surface (L1). Wavenumber domain plots of (**a**) $u_{x}$ at 110 kHz, 150 kHz, 185 kHz and 225 kHz, (**b**) $u_{y}$ at 110 kHz, 150 kHz, 185 kHz and 225 kHz, (**c**) $u_{z}$ at 110 kHz, 150 kHz, 185 kHz and 225 kHz.

Next, the 3D-FFT is employed to transform the 3D displacement data ($u_{x}(x,y,t)$, $u_{y}(x,y,t)$ and $u_{z}(x,y,t)$) into the frequency-wavenumber domain ($u_{x}(k_{x},k_{y},\omega )$, $u_{y}(k_{x},k_{y},\omega )$ and $u_{z}(k_{x},k_{y},\omega )$) and are shown in Figure 8a–c, respectively. In this work, frequency transformation is performed only on the data obtained from the top surface (L1). The size of the matrix used to store the 3D displacement data was 163 × 250 × 8000. Wavenumber plots at the frequencies 110 kHz, 150 kHz, 185 kHz and 225 kHz, are presented in the Figures. It is seen that both the S_0_ and the A_0_ modes appeared in the form of two concentric circular rings. The radius of the circles corresponds to the wave numbers at the respective frequencies. Wavenumbers of the S_0_ and A_0_ modes at the 150 kHz are obtained from the Peri-elastodynamic simulation and are 0.56 rad/mm and 0.187 rad/mm, respectively. A smaller circle corresponds to the S_0_ while the larger corresponds to the A_0_ mode. It is also observed that the energy of the modes at the frequencies 110 kHz, 185 kHz and 225 kHz are lower compared to that of at the 150 kHz. This is because most of the energy of the modes is concentrated around the excitation frequency (150 kHz). 

**Figure 9:**
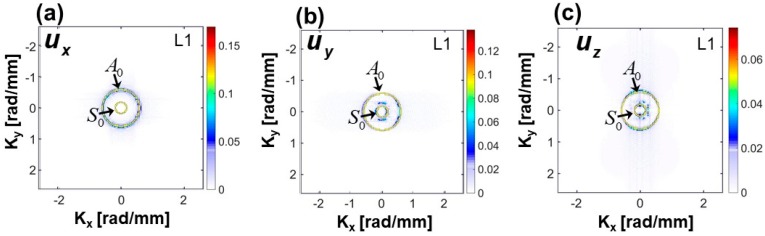
Comparison of theoretical and numerical (Peri-elastodynamics) wavenumber domain at 150 kHz: (**a**) $u_{x}$ at 150 kHz, (**b**) $u_{y}$ at 150 kHz, (**c**) $u_{z}$ at 150 kHz.

To verify the directional dependency of the Lamb wave propagation, 2D wavenumber plots ($u_{x}\left(k_{x},k_{y}\right)$, $u_{y}\left(k_{x},k_{y}\right)$ and $u_{z}\left(k_{x},k_{y}\right)$) at $\omega_{c}$ = 150 kHz, are obtained from the 3D FFT and were compared with those obtained from the theoretical predictions using Disperse software at 150 kHz. Theoretical wavenumber plot is superimposed on the numerically obtained wavenumber plots in Figure 9a–c. Good agreements between the numerical and analytical results are obtained. It shows that the Peri-elastodynamic can predict the dispersion relation of fundamental Lamb wave modes accurately in all directions. Therefore, the strong evidences discussed above demonstrate that the Peri-elastodynamics would be a potential tool to effectively simulate the Lamb wave modes for NDE and SHM applications.

## 6. Simulation of Wave Damage Interaction

**Figure 10:**
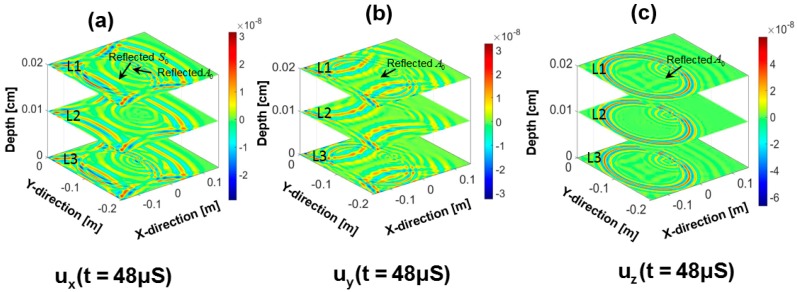
Time domain displacement waveform in a plate with a circular hole. (**a**) $u_{x}(x,y,t)$ at t = 48 μs, (**b**) $u_{y}(x,y,t)$ at t = 48 μs, (**c**) $u_{z}(x,y,t)$ at t = 48 μs.

**Figure 11:**
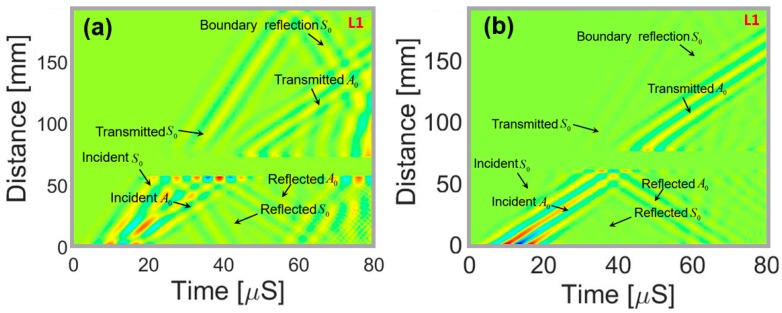
Space-time wavefield representations for top surface of the plate: (**a**) $u_{x}(x,y,t)$ for plate with a through-thickness hole (**b**) $u_{z}(x,y,t)$ for plate with a through-thickness hole.

The study of wave-damage interaction is an important part of wave propagation simulation. Thus, to demonstrate the feasibility, in this article, a simulation is performed on a plate with a circular hole as shown in Figure 2. The in-plane displacements in the simulated wave field demonstrate the reflection and transmission of the respective wave modes in three different layers across the depth. Figure 10a–c, show the displacement wave fields in the plate with circular hole after 48 μS. Only the vertical displacement pattern has the reflection from the hole in the wave fields at all the three layers. However, the reflections from the hole are observed in the wave fields with in plane displacements at the top and the bottom layers only. Figure 11a,b shows the 2D-FFT analysis of the $u_{x}(x,y,t)$ and $u_{z}(x,y,t)$ displacements at the top surface of the plate, respectively. Reflected and transmitted S_0_ and A_0_ wave modes are clearly visible but much stronger for $u_{x}(x,y,t)$ displacement in Figure 11a and weaker for $u_{z}(x,y,t)$ displacement in Figure 11b. Boundary reflection of the same modes are also clearly visible. Based on the simulation results, a PZT sensor at a distance of 45 mm from the center of the circular hole along any direction will be suitable to detect the damage in the PZT mounted plate-like structure. If the PZT sensor is placed towards the positive *X* axis from the hole, the transmitted wave signals will be received by the sensor but if the PZT sensor is placed towards the negative X axis from the hole, the reflected wave signal will be received. Based on the simulation results presented in Figure 10, it can be said that if a PZT with 1-3 and 1-1 polarization is used, any location is suitable to detect the hole with both transmitted and reflected wavefronts. Because the superposition of the transmitted and reflected wavefront will contain both the in plane $u_{x}(x,y,t)$ and $u_{y}(x,y,t)$ and the out of plane deformation $u_{z}(x,y,t)$ of the PZT attached to the plate.

Video S2 in the supplementary material shows the guided wave interaction with a hole in the plate through the $u_{z}(x,y,t)$ displacement of the layer L1 used in this article.

## 7. Perspectives and Peri-Elastodynamic Application to the MEMS Devices

Similar ultrasonic characterization of the plate and membrane-like structures are used in micro-electro-mechanical systems (MEMS). Characterization of MEMS requires an understanding of wave propagation and wave damage interactions with the fundamental Symmetric and Antisymmetric wave modes at their respective scales. For MEMS characterization, ultrasonic waves are generated by high frequency focused transducers or by Laser ultrasonic devices. It is a daring task to understand the ultrasonic signal generated from such inspections of MEMS devices and requires a virtual analysis of wave propagation in MEMS for their enhanced or more accurate characterization. NDE is a crucial step for their ensured functionality and fail-safe design of few specific plate-like MEMS. Such virtual NDE experimental scenarios for MEMS could be simulated using Peri-elastodynamic as proposed in this article. 

## 8. Conclusions

In this article, a numerical wave field computational tool called Peri-elastodynamics is proposed to simulate the guided waves in a plate-like structure with surface mounted PZT. Feasibility of the method is proved by simulating an SHM problem with PZT induced Lamb wave propagation in an isotropic aluminum plate. Fundamental symmetric (S_0_) and antisymmetric (A_0_) Lamb wave modes were generated. Further, their characteristics were investigated and compared with the theoretical predictions. Particle displacements due to S_0_ and A_0_ mode propagation were visualized through the vector-field plots across-the-thickness of the plate. Lamb wave modes simulated by the numerical technique were presented in the frequency-wavenumber domain and compared with those obtained from the analytical predictions. It can be concluded that if the process described in the paper is adopted meticulously, the Peri-elastodynamics can simulate the Lamb wave propagation accurately. The computational time for the SHM problem presented in this paper is approximately ~48 h but can be easily accelerated by implementing the parallel computing. In this article, no parallel computing facility was used. Based on the reported computation time in the literature, it is anticipated that with the proposed formulation the wave propagation simulation using Peri-elastodynamic will be time efficient. But it is difficult to comment however, if there has been a gain in the computation time compared to the existing methods, as a direct comparison of a similar problem solved using different method is necessary and it is left for the future research. Such numerical computation is valuable for the computational NDE of structures and devices, where experimentally studying the wave damage interactions are expensive tasks. In future, virtual wave propagation in structure and MEMS devices using the complementary tools will help avoid expensive experiments but extract the right wave feature to first characterize and then certify the materials and devices. 

## Figures and Tables

**Table 1:** Material properties.

Aluminum 6061-T6 Material Properties	
Density, ρ	2700 kg/m^3^
Young’s Modulus, E	69 GPa
Poisson ratio, ν	0.33

## Supplementary Materials

The following are available online at www.mdpi.com/1424-8220/18/1/274/s1, Video S1: Peri-elastodynamic simulation showing Symmetric and Antisymmetric wave in an aluminum plate. Video S2: Peri-elastodynamic simulation showing Symmetric and Antisymmetric wave interaction with a circular hole in an aluminum plate.
